# The case for better governance to mitigate against childhood obesity: the UK experience

**DOI:** 10.1186/s12916-023-03124-x

**Published:** 2023-11-09

**Authors:** BMC Medicine

**Affiliations:** The Campus, 4 Crinan Street, London, UK

Overweight and obesity, defined as abnormal or excessive fat accumulation that may impair health, are, for the most part, preventable health issues. Despite this, global obesity has nearly tripled since 1975, and overweight and obesity causes more premature mortality than does underweight. Shockingly, it is estimated that, globally, there were 39 million children under the age of 5 who were overweight or obese in 2020.

Childhood obesity is linked to a range of non-communicable diseases (NCDs), the risks of which increase depending on age of onset and duration of obesity. These NCDs include cardiovascular and metabolic diseases, cancers, and musculoskeletal disorders, all of which are linked to a higher risk of premature death and disability in adulthood. Given the significant healthcare burden, both at a societal and individual level, investment into mitigating childhood obesity is paramount.

The current societal expectation is for the individual to manage their own risks of obesity by moderating eating habits themselves. However, there is a *wealth of evidence* to suggest that this is neither feasible nor fair. This is especially the case when considering the time and resource pressures some parents encounter when purchasing food for their children or considering peer pressures some adolescents may face when buying their own food. Therefore, it is arguable that creating optimal food environments to mitigate against childhood obesity is a social responsibility, and this means better governmental input.

Three years ago, the UK government published an anti-obesity strategy. This included measures to end the promotion of foods high in fat, sugar, or salt (HFSS) by restricting volume promotions (e.g., multi-buy deals) and also to ban advertisements on HFSS before the 9 pm watershed. There is *strong evidence* to suggest that ending multibuy deals would be an effective measure to combat overconsumption as there is for *restricting advertising* on junk food to children. Multi-buy deals are more common for unhealthy foods and are linked to their increased sales. Furthermore, there is a clear causal and dose–response link between unhealthy food advertisements and childhood obesity. Despite the evidence, both of these measures have been delayed for another 2 years—with the current Prime Minister of the UK stating, regarding multi-buy deals, that “I firmly believe in people’s right to choose – and at a time when household budgets are under continuing pressure from the global rise in food prices, it is not fair for government to restrict the options available to consumers on their weekly shop.” This statement is at odds with the scientific consensus: cases of type 2 diabetes in children and adolescents have *risen faster in Britain* than anywhere else in the world, which Diabetes UK attributes to be in part due to deprived families being “pushed towards unhealthy options.”

Another opportunity to enhance anti-childhood obesity measures is provision of free school meals (FSMs) across primary school education, regardless of socioeconomic status. A third of a child’s daily food and drink intake is consumed during the school day, so there is a real prospect to leverage this from a public health stand. Access to FSMs has been shown to be *linked* to lower rates of obesity, better attainment of grades and attendance, and improvement of diet quality and food security. In addition, modeling studies have shown expansion of FSM provision expansion to be highly cost effective. For instance, if FSM were to be expanded to all children in England, regardless of family income (Universal Free School Meals; UFSM), the core benefits over a period of 20 years would be around £41.3 billion, compared to a total cost of £24.1 billion (including capital expenditure). This is a return of £1.71 for every £1 invested and includes increased saving on food costs for families of £22.5 billion, increased lifetime earnings and contributions of £18.5 billion, and other smaller cost savings to schools and to the NHS (obesity costs). UFSM has been rolled out across primary schools in four boroughs of London, which has already shown to be effective in reducing the prevalence of obesity. Hence, there is no reason why this should not be implemented immediately. However, despite this evidence, there has been no movement to extend access to FSMs- another opportunity missed.

Critics of better governance regarding public health initiatives have always labeled such interventions part of the “nanny state,” citing people’s right to choose. Yet recently, the UK government signaled plans to introduce a law to increase the legal age to buy cigarettes year on year to eventually phase out the habit, citing the decision as one which would tackle the leading cause of preventable illness and death globally. Given that obesity is fast overtaking smoking as the leading cause of preventable deaths, there should be lessons learnt from tobacco policy to create a better food environment to mitigate against childhood obesity. The UK government could learn lessons from Chile, whose government restricts marketing to children of HFSS and bans them from sale in schools. This has resulted in a *reduction of sugary drinks* sales by almost a quarter.

A good government should put the needs of its citizens first and foremost. Strong policies that would likely be effective in terms of reducing childhood obesity, improving public health and being highly cost-effective should be implemented as a matter of urgency. This should not be regarded as interference from the “nanny state” but rather creation of a better food environment within which individuals will lead healthier lives.

We are welcoming submissions on the topic of pediatric obesity, including those linked to food policy, to be submitted to our Pediatric Obesity and Diabetes Collection.

## Data Availability

Not applicable.

